# “Data makes the story come to life:” understanding the ethical and legal implications of Big Data research involving ethnic minority healthcare workers in the United Kingdom—a qualitative study

**DOI:** 10.1186/s12910-022-00875-9

**Published:** 2022-12-16

**Authors:** Edward S. Dove, Ruby Reed-Berendt, Manish Pareek, UK-REACH Study Collaborative Group, Laura Gray, Laura B. Nellums, Anna L. Guyatt, Catherine Johns, I. Chris McManus, Katherine Woolf, Ibrahim Abubakar, Amit Gupta, Keith R. Abrams, Martin D. Tobin, Louise Wain, Sue Carr, Kamlesh Khunti, David Ford, Robert Free

**Affiliations:** 1 Edinburgh Law School, University of Edinburgh, Old College, South Bridge, Edinburgh, EH8 9YL UK; 2 Department of Respiratory Sciences, University of Leicester, Leicester, UK; 3 Department of Infection and HIV Medicine, University Hospitals of Leicester NHS Trust, Leicester, UK

**Keywords:** Big Data, COVID-19, Ethics, Ethnic minorities, Healthcare workers, Public health, United Kingdom

## Abstract

The aim of UK-REACH (“The United Kingdom Research study into Ethnicity And COVID-19 outcomes in Healthcare workers”) is to understand if, how, and why healthcare workers (HCWs) in the United Kingdom (UK) from ethnic minority groups are at increased risk of poor outcomes from COVID-19. In this article, we present findings from the ethical and legal stream of the study, which undertook qualitative research seeking to understand and address legal, ethical, and social acceptability issues around data protection, privacy, and information governance associated with the linkage of HCWs’ registration data and healthcare data. We interviewed 22 key opinion leaders in healthcare and health research from across the UK in two-to-one semi-structured interviews. Transcripts were coded using qualitative thematic analysis. Participants told us that a significant aspect of Big Data research in public health is varying drivers of mistrust—of the research itself, research staff and funders, and broader concerns of mistrust within participant communities, particularly in the context of COVID-19 and those situated in more marginalised community settings. However, despite the challenges, participants also identified ways in which legally compliant and ethically informed approaches to research can be crafted to mitigate or overcome mistrust and establish greater confidence in Big Data public health research. Overall, our research indicates that a “Big Data Ethics by Design” approach to research in this area can help assure (1) that meaningful community and participant engagement is taking place and that extant challenges are addressed, and (2) that any new challenges or hitherto unknown unknowns can be rapidly and properly considered to ensure potential (but material) harms are identified and minimised where necessary. Our findings indicate such an approach, in turn, will help drive better scientific breakthroughs that translate into medical innovations and effective public health interventions, which benefit the publics studied, including those who are often marginalised in research.

**Supplementary Information:**

The online version contains supplementary material available at 10.1186/s12910-022-00875-9.

## Background

The COVID-19 pandemic has affected individuals and communities across the world, but some more than others. The disproportionate impact of the disease (caused by the SARS-CoV-2 virus) on minority ethnic communities and lower-income populations in dense, urban areas has been well documented in the literature [1–4]. What has been covered less extensively is the impact of the disease on healthcare workers (HCWs) within the same communities [5–9]. Are healthcare workers, and in particular ethnic minority healthcare workers within a given population, also disproportionately impacted by COVID-19?

Answering this question has been the aim of UK-REACH (“The United Kingdom Research study into Ethnicity And COVID-19 outcomes in Healthcare workers”) (see Additional file 1 for list of members of the UK-REACH Collaborative Group). Specifically, the aim of the study, through different work packages adopting various research methods, is to look at if, how, and why HCWs in the United Kingdom (UK) from ethnic minority groups are at increased risk of poor outcomes from COVID-19 (see more information at https://uk-reach.org/main/), with a view to reduce health inequalities and improve the long-term health outcomes of HCWs. Research to date from the study has indicated, among other findings, that in the UK, HCWs from certain ethnic minority groups have been at higher risk of COVID-19 than White HCWs, and that there are differences in home and occupational factors that affect COVID-19 risk between ethnic groups. For example, home factors associated with a higher risk of infection included younger age and living with other “key workers”. Occupational factors associated with a higher risk of infection included attending to higher numbers of COVID-19 patients, working in a nursing or midwifery role, reporting a lack of access to appropriate personal protective equipment (PPE), and working in hospital inpatient and ambulance settings [10]. The study has also provided evidence of limited HCW access to PPE during the pandemic, as well as ethnic differences in SARS-CoV-2 vaccine hesitancy among HCWs [7, 11].

Coincidental with better scientific understanding of if, how, and why HCWs in the UK from ethnic minority groups are at increased risk of poor outcomes from COVID-19 is the need to understand the ways in which *data* are harnessed to answer this overarching research question, and the ethical and legal implications such data linkage and use might raise. The UK-REACH project involves the use of a wide range of high-volume datasets and their linkage to healthcare data. This activity is likely to be considered by participants and stakeholders as sensitive and to raise a diverse set of ethico-legal issues. In the UK, while there are robust legal regimes in place governing (directly or indirectly) the use of one’s personal information, such as the UK General Data Protection Regulation (the UK GDPR), the Human Rights Act 1998, and the common law duty of confidentiality, to name a few, there is also, nevertheless, a notable level of mistrust and scepticism from a number of communities regarding the ways in which a variety of data are put to use in research projects. This is particularly the case among some ethnic minorities [11, 12]. Such concerns may be more pronounced in large-scale “Big Data projects”, given explicit consent serves less frequently as the legal basis for data processing as compared to, for example, processing on the basis of a task carried out in the public interest. Concerns have also heightened, we suspect, through recent examples of discrimination arising through use of patient data in the UK, such as the use of National Health Service (NHS) non-clinical data by immigration authorities from 2017 onwards to check on the legal status of individuals using the health service and residing in the UK [13, 14]. We were interested in exploring these issues from an ethical-legal perspective and considering the ways in which such important but potentially fraught work can be done in a way that is ethically, legally, and socially acceptable.

In this article, we present findings from Work Package 3 (WP3), the ethical and legal work package in UK-REACH. Through semi-structured interviews involving key opinion leaders in healthcare and health research, we sought to understand and address legal, ethical, and social acceptability issues associated with the large-scale linkage of professionals’ registration data and healthcare data, what we term “Big Data research in public health”, and to situate this within existing literature exploring patient and research participant views on use of their data for health research purposes as well as broader studies on the ethics of Big Data [15–18]. We consider the UK-REACH project a Big Data-driven research project in that it not only involves the use of significantly large amounts of data; it also involves data from a variety of sources (e.g. health, employment, professional registration) obtained at high velocity from multiple data custodians (in part due to the public health urgency of the pandemic). In what follows, we make the case that despite several significant legal, ethical, and social hurdles researchers face in making use of biomedical Big Data concerning ethnic minority HCWs, most importantly concerns about misuse and mistrust, the upfront adoption and implementation of a “Big Data Ethics by Design” approach can help assure (1) that meaningful engagement is taking place with historically marginalised communities and that extant challenges are addressed as robustly and as early on in the research as possible, and (2) that any new challenges or hitherto “unknown unknowns” can be rapidly and properly considered to ensure potential (but material) harms are identified and minimised where necessary. Our findings indicate such an approach, in turn, will help drive better scientific breakthroughs that translate into medical innovations and effective public health interventions, which benefit the publics studied, particularly those who historically have benefited much less (if at all) from research and public health interventions.

## Methods

Qualitative research was seen as the best approach to accomplish the objectives mentioned above—that is, to explore various views on the ethical, legal, and social implications of large dataset analyses/cohort studies—what we call Big Data research, and which raises its own distinct challenges [19, 20]—in a public health context such as the COVID-19 pandemic. Semi-structured interviews with participants (conducted together by the first two authors) allowed us to gather key opinion leaders’ perspectives in-depth and to explore with them real-life processes of working through ethico-legal challenges in Big Data research, particularly where HCWs are the study population. The goal of this project, as in many qualitative research projects, was not to obtain statistical representation, but rather to obtain theoretical representation [21] to reflect particular features of the sample population of key opinion leaders in healthcare and health research in the UK.

Prior to recruitment, ethics approval was obtained from the London-Brighton & Sussex Research Ethics Committee of the Health Research Authority (Ref No 20/HRA/4718). We then engaged in strategic sampling [21], working with gatekeepers, members of the UK-REACH research team, and the UK-REACH Professional Expert Panel (the public involvement group in UK-REACH), to identify individuals (≥ 16 years of age) (a) who could speak to ethico-legal and social issues in Big Data research in public health from different disciplinary and experiential perspectives, and (b) who had experience in healthcare and/or health research, or in health-related organisations (such as regulatory bodies or trade unions). Keeping in mind the project’s resource limitations, ease of access also drove the sampling strategy. Therefore, we used convenience sampling and, after the initial interviews, snowball sampling, to recruit further participants with the aim to be representative of various regions across the UK. Our target range was 15 to 20 interviews.

The average interview time was approximately 45 min. Interviews were held two-to-one, which in our view, led to a more relaxed, conversational form of interview, and audio-recorded via Microsoft Teams. In the midst of the COVID-19 pandemic, synchronous virtual platforms have become routine in qualitative health research [22]. The authors then checked the transcription against the recordings to ensure accuracy. Simultaneous to the cross-check process, the authors also anonymised the transcripts prior to data analysis to protect the confidentiality of participants.

Transcripts were manually coded using qualitative thematic analysis, which offers theoretical freedom and flexibility to yield rich and detailed, yet complex, accounts of data [23, 24]. The first two authors independently coded the transcripts and then proceeded to engage in discussions to identify commonalities in themes. The identification and development of themes was iterative; we referred back to the transcripts to provide support for the themes and our interpretation of their significance.

## Results

Of 36 individuals contacted, 24 responded and 22 agreed to be interviewed in English. These 22 individuals were situated across all four nations in the UK, reflecting different socio-legal and research environments. Twelve of the participants were male-identifying (54.5%), while 10 were female-identifying (45.5%). In terms of self-identified ethnicity, 36.4% identified as White British and the remaining participants described themselves as having other diverse ethnic backgrounds (see Table 2). In terms of national distribution, 15 participants were situated in England (68.2%), 2 were situated in Scotland (9.1%), 2 were situated in Wales (9.1%), and 3 were situated in Northern Ireland (13.6%). With respect to job role, 5 described themselves as academics (in the fields of public health and epidemiology, data science, and health and social sciences), 2 as representatives from professional organisations, 7 as doctors or medical officers, 2 as equality, diversity, and inclusion (ED&I) leads from regulators, 1 as legal professional (barrister), 3 as senior managers from NHS Trusts/Health Boards, and 2 as research nurses or midwives. The professional distributions are reflected in Table 1. Table 2 provides the specific country and interview ID of each participant.

**Table 1:** Job roles of interview participants

Job role	Total
Academic	5
Committee chairperson—professional organisation	2
Doctor/medical officer	7
Head of ED&I—regulator	2
Legal professional	1
NHS trust/health board senior manager	3
Research nurse or midwife	2
Grand total	22

**Table 2:** Ethnicity and job location of interview participants

Participant number/interview ID	Ethnicity	Job location in the UK
1	Indian	England
2	Jewish	England
3	White British	England
4	Black British	England
5	Black Caribbean	England
6	White British	England
7	Welsh	Wales
8	Indian	England
9	White British	England
10	White British	Wales
11	White British	Northern Ireland
12	Indian	Wales
13	White Irish	Northern Ireland
14	Pakistani	Scotland
15	White British	England
16	British Asian	England
17	Black American	England
18	White British	England
19	Indian	Scotland
20	Filipino	England
21	White British	Northern Ireland
22	Indian	England

In the remainder of this section, we unpack each of the four main themes identified, namely mistrust, data use and handling, community involvement in research, and the pivotal point for research in the UK.

### Drivers of mistrust

Participants raised issues of mistrust, within and beyond research. Indeed, almost every participant we interviewed identified mistrust, broadly speaking, as a significant barrier to research participation and realisation of the research endeavour more generally, in particular with projects that specifically seek to investigate disparities of health outcomes among ethnic minorities (be they HCWs or otherwise). Mistrust, as our participants identified it, was referred to in multiple ways, similar to those identified by Ho et al. in their work [25] on medical mistrust among women with intersecting marginalised identities, viz.: a lack of trust in the healthcare system felt by those who have experienced discrimination when receiving care; suspicion of the treatment provided to an individual’s racial or ethnic group by mainstream healthcare systems and health professionals; a lack of trust in healthcare organisations and in medical personnel; a lack of confidence in the medical system and in the intentions and work of medical professionals; and a tendency to distrust medical systems and personnel believed to represent the dominant culture. In our study, mistrust also referred to a lack of trust in the safe handling and use of one’s personal information, as well as the lack of trust in government more broadly, which may speak to the closer alignment in the UK between the state and the healthcare system, including research conducted within and connected to the NHS. The underlying sources of mistrust are multifactorial—they may be both actual and perceived—and might be a consequence of, among other things, long-standing marginalisation of certain communities in UK society or “institutionalised racism” as several participants phrased it, and unethical use of data by the UK Government and others that has led to discrimination in various forms. As one participant told us:

I think there is sadly some growing mistrust of the way in which the NHS safeguards information and that’s I think as a consequence of a number of events over the years. If you look at care.data [26], if you look at the Memorandum of Understanding between the Home Office, the Department of Health and Social Care and NHS Digital that was seeking to use information to look at people who might have been guilty of immigration offences, [participant lists other recent examples]…, people feel that trust is being eroded and trust in the doctor/patient relationship is being eroded, with the consequence of reducing health-seeking behaviour, making people reluctant to consult or seek healthcare when they should. I think that that is particularly likely to have an adverse effect on already marginalised and hard to reach populations such as refugees and asylum seekers, undocumented migrants, traveller populations, the homeless, etc. (Participant 6)

Although participants suggested ethnic minority HCWs might have greater trust in Big Data public health research than the general public due to more familiarity with its methods and putative benefits, others noted that insider knowledge of NHS systems and failings might in fact make them *more* sceptical of how their data will be used.

Another participant emphasised how direct experience of discrimination within the NHS also shaped levels of mistrust in HCWs:

…we identified the groups that were mostly affected by discrimination, and that is not just the experience of discrimination, but witnessing it, as well as anticipating discrimination. So avoiding going for training or promotion or different opportunities because you think—you’ve watched other colleagues be discriminated against. You see how the NHS has not addressed many of the issues. […] So there certainly is a lot of mistrust. (Participant 17)

These sources of mistrust, participants informed us, can in turn lead to downstream effects such as vaccine hesitancy—in the present context, COVID-19 vaccination [11, 27–29]—and deep concerns about harms—be they dignitarian, psychosocial, or otherwise—arising from research participation and use of one’s data. Given that many participants expressed concerns about harms arising from data misuse, it is not surprising that they also spoke about safeguards and mechanisms that could allow data to be used and handled appropriately, particularly as a way to overcome mistrust and establish and maintain a “social licence” for such research; that is, the ability to have ongoing community acceptance and support for the research endeavour. This led to our second main finding regarding the scope of appropriate data use.

### Components of appropriate data use

Participants suggested that organisational and technical safeguards to promote appropriate data use, and prevent misuse, are crucial to enable research participation and trust in the research endeavour. At the same time, several expressed to us that linkage and use of certain data categories—in particular data concerning ethnicity—ought to be treated with caution for several reasons.

Data protection law in the UK takes a binary and individual-focused (rather than group-focused) position: data must be “personal” and relate to an individual “data subject” to fall within the scope of legal protection [30]. If data do fulfil these criteria, and also involve health data or data concerning ethnicity, then they are considered “special category” personal data and heightened legal restrictions apply to the processing. If data do not fulfil these criteria, because they are sufficiently anonymous and thus not “personal”, then the legal framework by and large does not apply to any processing activity performed on such data. Participants had mixed views about this binary and relatively narrow approach in data protection law. Some felt that it was appropriate that anonymous data (if properly anonymised) should not be subject to an onerous legal framework given people’s interests in “their” data would be minimal if the data could no longer be traced back to them as an individual. Others, however, felt that the current approach to disapply data protection law when data are anonymised was insufficient and gave rise to concerns that one’s ethical—and legal and professional—duties would dissipate, if not disappear, despite the possibility of individuals’ ongoing interests in data they have contributed, anonymous or not, in particular as a member of an ethnic minority community. Moreover, aa participants stated, “true” anonymisation is difficult to achieve with data linkage involving multiple data points (e.g. NHS number, postal code, professional registration data, health outcomes data).

As other participants explained to us, even if data are anonymised in the legal sense [31], if researchers are going to use those data to produce an interpretation that then will be able to be transformed or extrapolated to a similar population group, then there is a kind of “moral obligation” to tell research participants (Participant 8), if not engage with them from the outset and prior to data collection and analysis. This moral obligation becomes even more pronounced when vulnerable populations and/or ethnic minority communities are involved. Otherwise, there is concern of a “slippery slope” that could break the trust that is in the research system, creating an ongoing cycle of mistrust that undermines health and community wellbeing. Participant 21 put it that “even though GDPR isn’t applying technically, […] we still need to be really careful with how that data is used and the conditions that are put around its transparency, who’s looking at it, how it’s disposed of and all those other things.” To this end, participants were broadly in support of the concept of *dynamic anonymisation*, meaning an ongoing watching brief and test of (re)identifiability that assesses what is identifiable by reference not just to what is identifiable in most contexts and vis-à-vis those to whom data are being or might be disclosed, but also to what is identifiable in conjunction with data which are readily available or may be available to anyone seeking to re-identify data [32].

Participants similarly emphasised access and purpose limitation as key elements of appropriate data use in Big Data research in public health. This includes, but is not limited to, ensuring that data are securely stored and can only be accessed by certain people. Likewise, participants stressed the value of helping people think beyond if data should be used at all in the research endeavour, to asking *how* the data should be used and for what purposes, with a view to delineating the kinds of appropriate purposes to which data ought to be collected and used. As one participant put it: “I think it’s just that sort of ethos of asking people, ‘what do you think?’ and ‘how do you think?’ because they can have more of a stake in it, because the question of ‘do you think…’ is going to get some ‘no’ answers. And then everything gets very messy” (Participant 10). Along similar lines, several participants noted that in the Big Data and health context, purpose limitation is not enough—and in any event is significantly challenged when purposes for data processing can be varied and extensive. What is needed, participants told us, is clear, up-front communication of the purposes for which data are being collected, used, and shared (even if they are extensive), along with consideration for potential downstream implications that could lead to concerns of stigmatisation or discrimination, especially when vulnerable, historically marginalised, or ethnic minority communities are involved. As one participant told us:

I think a lot more information could be put into – at the point of data collection – explaining what it will be used for. So I think you could easily at the point of data collection say, “We will use it for research purposes, for example this, this and this”. And that should, I think, if it’s explained properly, clear that off. But at the same time, what that looks like, again whoever’s got the data has to have a really good understanding of purpose and sharing, so what is research and what is beneficial research and how does it support things? Because I have some discussions where people – you could see that the researchers want to use it for something negative like, you know, “show me the ethnicity of this group of people” and their plans are to show, like, in a kind of discriminatory way that a certain group behaves in a certain way and I don't think that’s OK, because I think that then counteracts equalities legislation and human rights legislation, so not just for everyone, for any purpose, I think it should be for good reasons that are not discriminatory for example. And that’s the fear of people – ‘I'm going to give you this and you're going to use it for discriminatory purposes’ – so I think it should make sure that it doesn’t in those contexts. (Participant 5)

While a few participants considered existing legal and information governance frameworks as unduly onerous and inhibitive to efficient data sharing and use for important public health research, many other participants noted data use and sharing must take place in an environment where people can trust that their data will be appropriately safeguarded and not used in ways that will either surprise them or cause them harm. This accords with the National Data Guardian’s research and the project Understanding Patient Data, which suggest publics support an approach to data use that prioritises “no surprises”, and which focuses on sharing identifiable information only in line with peoples’ reasonable expectations of privacy [33–35]. It also accords, on a more conceptual basis, with the work of scholars such as Helen Nissenbaum, whose theory of “contextual integrity as privacy” postulates that social activity, such as health, occurs in contexts and is governed by context-relative norms, including informational norms, and whether contextual integrity is preserved or violated by a (new) system of practice is assessed by people’s reactions to it: protest, acceptance, expressions of scepticism and mistrust, or otherwise [36]. Moreover, trust in researchers and in the healthcare system to protect and promote peoples’ interests as data subjects is fostered by *demonstrating* trustworthy behaviour, including through use of robust input mechanisms (e.g. data security, explaining the purpose clearly and honestly, ongoing engagement with data subjects) and robust output mechanisms (e.g. access limitations, data storage limitation periods, dissemination of research findings that do not exacerbate existing inequalities).

Finally, participants also raised concerns about data categories that, while important in Big Data research in public health, ought to be treated with caution for quality-related reasons. Specifically, this related to the category of data concerning ethnicity. Participants observed that such data may be incomplete and invalid, in part “because a lot of people are reluctant to divulge their protected characteristics” (Participant 6) and because the underlying input elements that comprise the category of ethnicity may differ from one dataset to the next (e.g. different terms for self-identification and ways of coding responses). Participant 7 was even more direct: “…ethnicity data is pants, it really is bad, and I still don’t think we have a really good answer of how we collect it, what the categories should be, and what the basic demographic comparators are […]”.

Nonetheless, participants did not suggest that because of these quality-related problems, research investigating health disparities among ethnic minorities is infeasible; they did suggest, however, that the input and output might need to be treated with caution. Thus, some spoke of the need for particular “granularity” with these data. Participant 20 put it thus: “In terms of gathering ethnicity, for me it’s not enough yet because it’s still very broad isn’t it? It especially shows in this pandemic that data in terms of collecting the correct ethnicity needs to be kind of meant in a way that’s much more clear.” This participant noted that different organisations have different boxes or ways of collecting data concerning ethnicity, and this can generate problems of data quality and being able to appropriately self-identify. Others noted a “small numbers problem” (Participant 10), meaning that a research project may involve ethnic minority HCWs and some may be worried about the level of disclosure that might happen if personnel data were to be linked to their health records.

### Improving community involvement in research

A significant number of our participants stressed the importance, with direct relevance to the above themes of mistrust and elements of appropriate data use, of involving communities in research. In other words, researchers—and funders and sponsors—ought to consider ways in which both participants and the wider community can be better engaged to encourage participation in research, and the direction of that research, that in turn is of benefit for that community [37].

Participants spoke, for example, of co-creation and co-production in Big Data research in public health, by which they meant deeper involvement in actively involving historically marginalised groups, involving them in the research design at the very start, and involving them all the way through the process. One participant spoke of the importance of maintaining a “social licence” in research (see also [26]), meaning:

…the idea that you behave in a trustworthy and transparent enough way and you put in additional safeguards that are kind of co-designed with potential participants to end users, to show you’re going above and beyond the regulation, because you really care about showing that you can be trustworthy. And I think that’s probably the way to go rather than just say ‘We’re sticking to the rules, it’s fine’. (Participant 18)

Similarly, Participant 17 spoke of the importance of “levelling the playing field in terms of the way that we engage in research to address some of the barriers, to identify what they are, to build the trust that’s necessary with communities, including healthcare workers.” This participant emphasised the benefit of using participatory action approaches and models of co-production [38] from the very beginning of the research endeavour.

Participants also mentioned the ethical values of reciprocity and benefit sharing in Big Data research in public health, particularly where ethnic minority communities (including HCWs) are involved. Participant 17 expressed this model of reciprocity as part of an ethos for researchers across the research lifecycle, asking themselves, “Are we giving back?” Others spoke of the link between cost—in particular, time and effort from participants to participate in the research project—and perceived benefits, and the need to engage with participants to explain the expected benefits from research. Participant 5 spoke of time as being a “huge barrier” for HCWs participating in research, and lots of research being “quite poor in explaining the purpose and what it’s going to be used for and how it will help things generally”; for them, researchers have an obligation to put effort into explaining the tangible and intangible benefits that research participation might bring, and how involvement may lead to positive outcomes for the community in which they are situated.

Finally, participants discussed the importance of being culturally aware, and in particular, having “intersectional awareness”, meaning understanding that individuals’ social and political lives are shaped by multiple axes of social division (such as race, socioeconomic status, gender, etc.), which interact and influence each other to create modes of discrimination and privilege [39, 40]. Participant 1, for example, discussed how “it is all about knowledge”, by which they meant culturally appropriate knowledge and awareness of the ways in which communities experience stigmatisation and marginalisation, and how in turn that affects their engagement with research and perceived trust or mistrust. For research projects similar to UK-REACH, participants informed us that it is crucial that sensitivity be deployed in working around inequalities regarding health outcomes stratified along categories of ethnicity, while maintaining the principle of being inclusive and also looking at intersectionality, where ethnicity may intersect with other characteristics, including socioeconomic status, gender, and geographic location.

In terms of practical approaches to being culturally aware and attuned to intersectionality, some participants mentioned that researchers ought not to shy away from looking at discrimination in a lot of detail, including structural discrimination and interpersonal discrimination. This entails actively listening to and engaging with the communities involved in the research—including HCWs themselves—and listening to concerns regarding historical issues of discrimination that had happened; the unaddressed issues, specifically around racism and around immigration policies; and the challenges that arise at the intersection between ethnicity and race, as well as potentially immigration status, as there may be inequalities that emerge when these are looked at together as opposed to singly. As one participant told us, HCWs can find it frustrating that researchers can “forget that they’re from a community. They talk about, you know, these studies that are sort of focused around the workplace, that forget that they are bringing all of their social experiences to the workplace” (Participant 17).

### A pivotal point for research in the UK

A final theme to emerge from the interviews concerned the future of research in the UK in the midst of a changing landscape. Participants spoke about COVID-19 as a catastrophe for the world, as all pandemics are. But they also spoke about COVID-19 as an opportunity to both shine light on longstanding inequities and inequalities in society, as well as an opportunity to demonstrate the value of Big Data research in public health, not to mention artificial intelligence and Big Data analytics more generally (of which the UK is perceived as one of the world leaders). At the same time, in the midst of the UK’s rather chaotic withdrawal from the European Union (EU) and ongoing political and economic uncertainty, there is some trepidation about whether the UK can continue to exert influence in establishing guidelines and rules for research, and remain a global leader in research and innovation [41].

As one example of how research is at a pivotal point, a participant mentioned that COVID-19 research could demonstrate that past practice of linking datasets for a single project and then destroying the datasets is “just a nonsense” (Participant 19). This participant suggested that this approach means that realistically any project takes several years just for governance aspects alone, and it also means that researchers cannot do things in a timely and responsive way if there is a pandemic. Participants, though relatively confident that the landscape for life sciences looks positive in the UK going forward, were less confident in terms of sustained large-scale funding investigating social influences of health and health inequalities within the UK.

Participants also discussed the benefits and drawbacks of opt-in versus opt-out consent as an ethical basis for research participation (rather than as a legal basis for data processing), with concerns especially about the practicality of opt-in consent. In the views of several participants, one of the strengths of analysing linked data is that it is one of the most robust ways of being able to study the health of socially excluded, marginalised groups across an entire population—not only ethnic minorities but also, for example, homeless people, people with severe mental illnesses, and migrants. Some participants expressed concern that if researchers needed to obtain each individual’s permission to use their data in public health research, then the UK would revert to a situation where those groups continue to be ignored and excluded, with ongoing health disparities and inequities. For these participants, then, the future landscape for research in the UK needs to recognise the harms of making it difficult for people to engage with research by requiring opt-in consent, and how much risk each individual participant faces versus the potential for real benefit to them and to UK society. At the same, it is important to recognise the potential harms that come with opt-out consent in the context of data-driven research, in particular diminished individual choice in use of one’s data (whether anonymised or not). Our participants in turn acknowledged that an opt-out consent approach could be seen as acceptable in Big Data research in public health with the proviso that public participation, transparency, accountability, and a robust case for the public and social benefits of using the data are firmly in place through well-established mechanisms (e.g. independent review committees), and that there is ongoing demonstration of ethical and legal responsibility undertaken by the research team.

Other participants noted the pivotal point for the research community in a post-Brexit environment, as well as the “tension” between commercial organisations that want to exploit NHS and other kinds of health data, and individuals who want to keep data about them secret. Participants surmised that the UK Government’s focus, especially following Brexit and the COVID-19 pandemic, will be primarily on promoting innovation and entrepreneurship in the UK. This will create challenges regarding compliance with extant data protection law and human rights law. Participants were not optimistic about these legal regimes being adequately protected going forward, and that presenting innovation and research protections as antagonising forces (as has been seen in UK Government pronouncements recently) is not a wise approach. For these participants, there was concern there would be a “drift” away from the protection of individuals' and communities' interests towards commercial (and other) organisations’ easy ability to exploit personal data and in consequence, exacerbate mistrust among ethnic minority communities.

Finally, participants also expressed hope that coming out of the pandemic, funders, and sponsors, not to mention the government, would provide more space to ethnic minority researchers (in terms of funding calls, media, and so on) and allow their voices to be amplified. In turn, the hope is that by continuing to shine a light on existing health disparities, more research can be conducted that leads to knowledge and innovation that can potentially deliver better services and do something about those health disparities on the ground. Here, participants emphasised the power of data, as Participant 4 put it:

….what we are seeing with COVID-19 is that the publications of the research, the publication of the data, is validating what people already knew, so if you already know something and you just know it as knowledge because you know that as a Black woman you would not have as much clout behind whatever you say – and I speak from my position – whereas a Black man that when you're walking along the street people will tend to cross the road if it’s rather dark because they would assume that you may be aggressive or violent, you'd know that because that’s your lived experience, but when it has been validated by figures then it makes it more powerful. Data makes the story come to life.

## Discussion

Our analysis demonstrates that among our interview participants, there is broad support for Big Data research in the field of health and public health, and recognition of the benefits it can offer. Our analysis also shows, however, that this support is contingent on at least three core clusters being addressed squarely by researchers and, more broadly, stakeholders that help support research (e.g. funders, sponsors, journals, ethics committees, data access committees).

First, our participants identified mistrust—of the research enterprise, of researchers, and of benefit to those who participate in research—as an ongoing significant barrier to the success (and public acceptability) of such projects. This aligns with research from projects such as Understanding Patient Data, which has highlighted the varying degrees of public trust in research that makes use of patient data, and how that trust is in part contingent on the proposed data use and the entities with whom such data will be shared, not to mention the measures put in place to safeguard those data [42]. Our participants suggested that mistrust in relation to Big Data research is not necessarily a product of the research itself, but rather a characterisation of the wider context and cultural narratives about data misuse, and mistreatment of communities more broadly. For example, such issues take particular form at the intersection of ethnicity and healthcare work, with mistrust surrounding data potentially affected by experiences of structural racism within the NHS (i.e. individuals’ experience of being an ethnic minority HCW). Where Big Data projects seek to use employment, professional registration, health, and a variety of other data categories, the historic mishandling of data by healthcare-affiliated bodies (e.g. the NHS, the General Medical Council) may lead to increased mistrust in the study findings or that data will be handled appropriately, particularly if these bodies are also involved in some form with the project. For Big Data projects to mitigate these concerns of mistrust, and to garner sustained trust by publics from the design stage, researchers ought to identify the drivers of mistrust relevant to that particular project and use an ethically informed approach to address and overcome mistrust. This is an approach we have outlined in more detail in our earlier article [32]. Such an ethically informed approach, which we term a “Big Data Ethics by Design” approach, incorporates the appropriate management and handling of data, as well as the direct and close involvement of communities with interests pertinent to the research project, right from the outset. This approach holds that ethical values and principles in Big Data health research projects are best adhered to when they are *already integrated* into the project aims and methods at the design stage. Adopting this approach, we argue, will help such projects become more “successful” in their endeavours (regardless of what the specific research findings might be).

Second, we found that existent mistrust by putative participants (and publics) is not necessarily an insurmountable barrier and that, when designed and undertaken in an ethical manner, Big Data research projects can establish confidence in their endeavours and overcome mistrust.

The first element of this, based on our findings, is to ensure that data gathered as part of a project is handled and used appropriately and sensitively. This includes ensuring the data use is lawful, the project has been reviewed favourably by an ethics committee, and that the research team has identified, minimised, and justified any potential risks or harmful outcomes. Where research focuses on ethnicity, our participants also suggested that the data gathered ought to be interrogated as to their quality, given the different ways in which ethnicity data are categorised and collected not only within a country, but also across sectors and systems (e.g. local authorities, the NHS, census data).

The second element is ensuring meaningful involvement of communities in research, in a manner that is sensitive to the particularity of the community or group being studied, and is attuned to the nature of communication or involvement that would be required to facilitate their proper involvement. Our participants recommended different ways meaningful involvement could be set up, which will necessarily be contextual and depend on the specific nature and objective of the project, as well as the community setting in which the research is taking place. These approaches may include co-creation/co-production of the research design, instantiation of the reciprocity principle—what Maiter and colleagues define as an “ongoing process of exchange with the aim of establishing and maintaining equality between parties” [43], and benefit sharing arrangements for knowledge exchange, capacity building, and bringing research outputs back into the communities being studied. From our own UK-REACH experience with the PEP, online meetings held outside of regular working hours helped facilitate the involvement of busy HCWs and increased their willingness and ability to input advice to the project. We also found that partnering with ethnic minority HCW organisations from the outset (e.g. Filipino Nurses Association United Kingdom, British Association of Physicians of Indian Origin, Sudan Doctors Union UK, Association of Pakistani Physicians of Northern Europe) helped build trust from within these communities. Some of our participants suggested in addition that these different forms of meaningful involvement could also be a means to help overcome hesitancy about data sharing by different stakeholders. That is, the involvement of communities and the securing of the social licence engendered by the research project—demonstrated through ongoing support and evidence of public acceptance—could help support efforts to responsibly share data, too, both within the project itself and following its completion to help support future research projects. Such sharing would, we hasten to add, have to be done in a way that accords with peoples’ reasonable expectation of privacy, particularly if the data remain identifiable. Ascertaining such reasonable expectations of privacy and appropriate data use itself would be best accomplished through robust public involvement; this might include, for example, workshops or citizens juries with members of the public who are representative of the population of a local area or region to better understand what might impact the creation of reasonable expectations and the role of acceptability in the creation of those expectations [for evidence of the successful use of citizens juries to explore public views on a range of health issues, see e.g. 44, 45].

Finally, our participants noted that the UK is in a period of flux as the country struggles to chart its own path post-Brexit and recover from the COVID-19 pandemic and larger macroeconomic forces. Our participants were clear that data-intensive science will continue to feature prominently in research in the biomedical and public health domains, including those harnessing Big Data analytics. This, our participants felt, was on the whole a positive evolution and one that would lead to better understanding of health and possibly more targeted and effective public health interventions. As noted above, however, the path forward in the UK for Big Data research is not written in stone, and nor is it free from risk. The country may yet stumble coming out of the pandemic recovery and may find itself facing “regulatory headwinds” as it seeks to develop new rules for research now that it is untethered to the EU. Separate from the specific content of any future regulation, our analysis demonstrates that Big Data research needs smart and effective regulation to enable it to thrive and sustain public trust. This includes a robust data protection regulatory environment as we believe currently exists in the UK, and smart and effective regulation that ensures private sector (commercial) organisations make use of patient and health data in ways that accord with people’s reasonable expectations of such use. This future regulatory environment, however, also requires clearer information governance guidance for data custodians that can enable data to be used and shared in a responsible manner [46]. To date, as many of our interview participants noted, a lot of data are *not* shared—even when they can and should be done so in a safe and effective manner (through e.g. secure data environments/trusted research environments). This is not so much due to data protection laws that prohibit sharing. Rather it is more a consequence of information governance rules and processes shaped by data custodians who feel reluctant to share data because of a “culture of caution” and—we would argue—undue over-caution with regards to perceived risks. As past research has indicated, such an overly strict and conservative approach not only is unwarranted in most instances, but it may actually undermine the intentions of patients and publics who wish their data to be shared responsibly for health research and to drive scientific breakthroughs and effective public health interventions [47].

Big Data research, our analysis demonstrates, also needs to be attuned to issues of justice and equity (as well as equality, diversity, and inclusion), and to this end, we recommend that funders provide significantly more opportunities (in training, in funding, and so on) for ethnic minority researchers and to enable their voices to be amplified. We would also recommend that funders develop schemes that focus research attention on health disparities and health issues that significantly impact minority communities, and as part of this, build in funding that helps those research projects develop knowledge and innovation that can potentially deliver better services and address health disparities on the ground.

### Recommendations to instantiate “Big Data Ethics by Design”

Based on the foregoing discussion, we propose several practical recommendations to give effect to a “Big Data Ethics by Design” approach, focusing on the legal and ethical dimensions, that can help researchers  gain and maintain trust in their research (Table 3) (see also [32, 48] for related discussion of the policy implications of Big Data health research studies such as UK-REACH). We think this approach complements other emerging approaches such as ethics parallel research or embedded ethics [49, 50] in that it not only ensures research teams consider these dimensions right at the design phase of their study and then throughout the study’s lifecycle and makes them an integral part, but it also places explicit focus on the Big Data-specific dimensions of a study in the area of health or public health, ensuring engagement, transparency, and sensitivity to factors of trust and mistrust in data usage are addressed robustly. In what follows, our recommendations align with and build on existing work on the ethics of Big Data [15–18]. We purposely do not intend them to be prescriptive or overly specific; this is so that they are generalisable beyond Big Data research involving ethnic minorities or HCWs, and have greater purchase for all sorts of projects seeking to harness the power of data to yield new insights into health and health outcomes in a given community or population.

**Table 3:** Recommendations to give effect to a “Big Data Ethics by Design” approach

Recommendation 1	To mitigate concerns of mistrust, and to garner sustained trust by publics at the design stage, researchers ought to identify the particular drivers of mistrust relevant to that particular project and use an ethically informed approach to address and overcome mistrust. This should involve an in-depth mapping exercise, iteratively conducted over the beginning stages of the research endeavour (i.e. from the design and early-implementation stages) to identify potential drivers of mistrust. This mapping exercise in turn ideally should involve both desk-based research and in-person engagement with the communities being studied (e.g. information discussions, focus groups, interviews) to ensure that all potential drivers are identified. This also ought to include engagement with HCWs’ employers (e.g. NHS trusts, NHS Health Boards) and their professional regulators (e.g. General Medical Council, Nursing and Midwifery Council)
Recommendation 2	Research teams should be mindful of relevant vulnerabilities of individual participants in their study, as well as groups of participants (e.g. sectors of HCWs, ethnic minority communities) and the risk of group harms such as stigma, mitigating these where appropriate. This also includes the need for an “intersectional awareness”, i.e. that individuals’ social and political lives are shaped by multiple axes of social division (such as race, socioeconomic status, gender, etc.), which interact and influence each other to create modes of discrimination and privilege. This awareness can be enhanced through the mapping exercise (Recommendation 1) as well as through engagement with organisations representing the groups of participants/communities involved in the research (e.g. a nurses or doctors union for a particular ethnic minority community)
Recommendation 3	Research teams should ensure there is meaningful involvement of communities in research, in a manner that is sensitive to the particularity of the community or group being studied, and is attuned to the nature of communication or involvement that would be required to facilitate their proper involvement. We suggest that this involvement can take different forms, participatory action approaches, and models of co-production. The form will necessarily be contextual and depend on the specific nature and objective of the project, as well as the community setting in which the research is taking place. This can range from citizens juries to workshops to inclusion of a community panel or advisory board within the research team. Research teams should engage with and encourage participation of individuals with morally relevant interests as key stakeholders. The makeup of the stakeholder groups should be reviewed on an ongoing basis to ensure those with morally relevant interests are included at all stages and afforded a meaningful opportunity to influence decision-making and the direction of the study. This meaningful involvement can help assure a watching brief is kept on the relevant ethical values at play, in turn helping assure that the study’s activities continue to be proportional to the benefit sought, and to identify any new issues of ethical concern that may arise
Recommendation 4	Where data and datasets are deemed to be anonymised according to data protection law and commonly accepted standards (e.g. those promulgated by a national data protection authority), studies should continue to consider what legal and ethical obligations arise even after anonymisation has been achieved. This “dynamic” approach to anonymisation includes keeping a watching brief on what is required to ensure the data remain anonymous throughout the lifecycle of their use in a study and in any subsequent, downstream uses, as well as a watching brief on confidentiality of the data, risk of re-identification, human rights, equality/anti-discrimination protection, and other relevant interests
Recommendation 5	Research teams should ensure that in the collection, storage and dissemination of research findings, there is no discriminatory impact (directly or indirectly) on the participants and communities involved in their study. Involvement of a panel of the particular community or communities involved during the research findings stage of the research, and prior to any public release or dissemination, ought to be considered. The ethical values of reciprocity and benefit sharing suggest that researchers ought to be engage communities from the outset to identify the tangible and intangible benefits that research participation might bring, and how involvement of those communities may lead to positive (health) outcomes for the community in which they are situated

### Limitations

Our qualitative research study in UK-REACH has some limitations. First, we interviewed only individuals we identified as “key opinion leaders” (which we took to mean academics, information governance officers, senior HCWs, and scientists/researchers) in healthcare and health research. This therefore excluded perspectives from other critical actors such as participants in other domains who could offer additional insight into Big Data research in public health, including citizens among different ethnicity minority community groups across the UK. As our research focused on the perspectives of experts in the field of medicine and health research, the views expressed may not be representative of those in other research contexts. However, some participants felt more comfortable discussing other areas of research, such as social science research in health, and therefore our findings may have resonance beyond the confines of Big Data research in public health as we define it. Second, it is possible that given several participants were involved indirectly in UK-REACH, they may have had more positive views of the nature of the study and research more generally, and as such, some of the findings here may not be representative of the broader population. Third, the framing of our qualitative study and several of the interview questions may have meant that those who had a particular story to tell or a particular interest in ethico-legal issues in Big Data research in public health, as well as research involving ethnic minority communities, were more likely to share their insights of perceived barriers and possible work-arounds. Finally, although qualitative data provide valuable insight into conceptually nuanced topics such as the ethical and legal issues associated with Big Data research in public health, our findings are not externally generalisable per se. This said, we believe our thematic findings can generate “theoretical generalisability” [21]; in other words, they can be used to generate hypotheses and queries for subsequent investigation.

## Conclusions

Our findings indicate that mistrust is a significant feature in Big Data research in public health that involves ethnic minority HCWs. We think this speaks to broader concerns of mistrust within society and marginalised community settings. The “bigness” of Big Data can exacerbate these concerns. However, our findings also indicate that ethically informed approaches can be crafted that help mitigate or overcome mistrust and establish greater confidence in data linkage and use. Big Data research in public health will likely continue to grow in importance and not just in relation to COVID-19 alone. Assuming that is the case, establishing confidence in the way such studies are designed and implemented will be a crucial task of all stakeholders involved in the research enterprise. For research that focuses on or includes minority communities (including ethnic minority communities), attention to equality, diversity, and inclusion considerations is paramount. A “Big Data Ethics by Design” approach can help assure that meaningful engagement is taking place and that extant challenges are addressed, and also help assure that any new challenges or hitherto unknown unknowns can be rapidly and properly considered to ensure potential (but material) harms are identified and minimised where necessary. Such an approach, in turn, can help drive better scientific breakthroughs that translate into medical innovations and effective public health interventions, which benefit the publics studied.

## Supplementary Information

**Additional file 1.** UK-REACH Collaborative Group.

## Data Availability

Anonymised datasets analysed during the current study are available from the corresponding author on reasonable request.

## Abbreviations

EU: European Union
GDPR: General Data Protection Regulation
HCW: Healthcare worker
PEP: Professional Expert Panel
PPE: Personal protective equipment
UK: United Kingdom
UK-REACH: The United Kingdom Research Study into Ethnicity and COVID-19 Outcomes in Healthcare Workers
