# Supplementary material for: “Data makes the story come to life:” understanding the ethical and legal implications of Big Data research involving ethnic minority healthcare workers in the United Kingdom—a qualitative study
Source: BMC Med Ethics. 2022 Dec 16;23:136. doi: 10.1186/s12910-022-00875-9 (PMC9756740; doi:10.1186/s12910-022-00875-9)
Supplement: Supplementary file 1 — Additional file 1. UK-REACH Collaborative Group. [file 12910_2022_875_MOESM1_ESM.docx]

**Appendix 1: UK-REACH Collaborative Group**

Manish Pareek^2*^, Edward Dove^1*^, Laura Gray^3^, Laura B Nellums^4^, Anna L Guyatt^3^, Catherine Johns^3^, I Chris McManus^5^, Katherine Woolf^5^, Ibrahim Abubakar^6^, Amit Gupta^7^, Keith R Abrams^8^, Martin D Tobin^3^, Louise Wain^3^, Sue Carr^9^, Kamlesh Khunti^10^, David Ford^11^, Robert Free^12^

^1^ School of Law, University of Edinburgh, Edinburgh, UK

^2^ Department of Respiratory Sciences, University of Leicester, UK; Department of Infection and HIV Medicine, University Hospitals of Leicester NHS Trust, UK

^3^ Department of Health Sciences, University of Leicester, UK

^4^ Division of Epidemiology and Public Health, School of Medicine, University of Nottingham, UK

^5^ University College London Medical School, UK

^6^ Faculty of Population Health Sciences, University College London, UK

^7^ Oxford University Hospitals NHS Foundation Trust, UK

^8^ Centre for Health Economics, University of York, UK

^9^ General Medical Council, UK

^10^ Diabetes Research Centre, University of Leicester, UK

^11^ Population Data Science, Swansea University Medical School, Swansea, UK

^12^ Department of Respiratory Sciences, University of Leicester, Leicester, UK; NIHR Leicester Biomedical Research Centre, University of Leicester, Leicester, UK

^*^ Dove and Pareek have authorship status.
